# Structural and Histochemical Alterations in the Aortic Valves of Elderly Patients: A Comparative Study of Aortic Stenosis, Aortic Regurgitation, and Normal Valves

**DOI:** 10.1155/2016/6125204

**Published:** 2016-09-25

**Authors:** Katsutoshi Miura, Hideki Katoh

**Affiliations:** ^1^Department of Health Science, Pathology and Anatomy, Hamamatsu University School of Medicine, Hamamatsu, Japan; ^2^Division of Cardiology, Department of Internal Medicine, Hamamatsu University School of Medicine, Hamamatsu, Japan

## Abstract

The aim of this study was to reveal the pathogenesis of aortic stenosis (AS) and regurgitation (AR) by comparing differences in mechanical and biochemical alterations. We applied scanning acoustic microscopy (SAM) to measure the speed of sound (SOS) through valves to estimate the elasticity and monitor sensitivity to protease treatment, as the SOS is correlated with the stiffness of materials, which is reduced after digestion by proteases. The fibrosa of both the AS and AR groups were stiffer than the fibrosa of the normal group. The AR group displayed significantly stiffer fibrosa than the AS group, with the exception of calcified areas. The AS group showed significantly decreased SOS values following protease digestion, whereas the AR showed little reduction. The AS group presented type III collagen in the fibrosa and the ventricularis. In the AR group, both type I collagen and type III collagen coexisted in the fibrosa and the ventricularis. Upon immunostaining for advanced glycation end-products, the AS group showed sparse, weak staining, whereas the AR group presented a strong, band-like positive reaction in the fibrosa. In conclusion, tissue remodelling associated with damage and repair is associated with AS pathogenesis, whereas static chemical alterations with slow collagen turnover induce AR.

## 1. Introduction

During an average human life span, heart valves open and close approximately 3 billion times and withstand various mechanical stresses, including fluid shear stress and stretch bending. Aortic valves (AVs) are composed of living tissues and hold the ability to repair and remodel in response to damage. However, structural changes in AVs due to ageing can be accompanied by biochemical and functional alterations, causing aortic stenosis (AS) [1] or aortic regurgitation (AR) [2].

Resected AVs from elderly patients undergoing AV replacement surgery often show the following two distinct morphological characteristics: (1) thickened leaflets with nodular calcification and (2) transparent thin leaflets with rare calcification. The former corresponds to valves typical of AS, and the latter corresponds to valves typical of AR. The aim of this study was to reveal the pathogenesis of AS and AR by comparing differences in mechanical and biochemical alterations.

Investigations of the morphological and functional changes that occur in AVs during ageing require basic histological and biological knowledge. AVs consist of three distinct layers, the fibrosa, spongiosa, and ventricularis, which are composed of extracellular matrix that is rich in collagen, proteoglycans, and elastin. The elastic fibres of the ventricularis on the flow side of the valves are radially aligned and elastic. They extend when the valves open and recoil when the valves close. Proteoglycans in the middle layer, or the spongiosa, function as cushions for absorbing tension and friction. The fibrosa contains circumferentially oriented type I and type III fibrillary collagen fibres, which confer stiffness and strength to the valves [3, 4].

Scanning acoustic microscopy (SAM) can be performed to calculate the speed of sound (SOS) through tissues, and plotting these data sets allows users to create images of the tissue under examination [5, 6]. The relationship between the SOS and the elastic bulk modulus of a liquid-like medium is given by the Newton–Laplace equation, as follows:

(1) $$\begin{array}{c} c=\sqrt{\frac{K}{\rho }}, \end{array}$$

where *c* is the SOS (m/s), *K* is the elastic bulk modulus (N/m^2^), and *ρ* is the density (kg/m^3^). Thus, the SOS increases with the stiffness (the resistance of an elastic body to deformation by an applied force) of the material, whereas it decreases with the density. Excluding bones (1.750 g/cm^3^) and fats (0.9094 g/cm^3^), the average soft tissue density is approximately 1 g/cm^3^ [7], which is the same as water, and the SOS through soft tissues is strongly correlated with their stiffness.

We previously reported soft tissue elasticity [8–13], which reflects the flexibility or stiffness of various organs. The stiffness of each layer of AVs can be estimated by SOS values. Moreover, SAM is a useful tool to evaluate chemical alterations of tissues [8]. The SOS reduces after protease treatment, and its degree corresponds to protein cross-linkages [8]. These structural alterations depend on the biochemical changes of protein components, which can be detected by immunohistochemical (IHC) analyses. We used antibodies against collagen types I and III, lysyl oxidase (LOX), which catalyses cross-linking between collagen and elastin for stabilization [14], and advanced glycation end-products (AGEs), which are nonenzymatic modifications of proteins that proportionally appear with ageing [15].

In this study, we used formalin-fixed, paraffin-embedded (FFPE) sections of AVs of elderly patients, including those exhibiting normal and diseased AR or AS, to evaluate differences in mechanical and biochemical alterations.

## 2. Materials and Methods

### 2.1. Tissue Acquisition and Preparation

AV leaflets were obtained from surgical specimens of elderly patients undergoing AV replacement for AS or AR (Table 1). As controls, normal AV leaflets from autopsy cases without cardiac and metabolic diseases were used. We cut each leaflet into several sections and selected representative sections from each leaflet. For each case, formalin-fixed, paraffin-embedded sections were made for histological diagnosis. Decalcification of tissues was performed using a mixed solution of 9% formic acid and 3% HCl, wherever necessary. Samples were taken vertically through the cusp and sinus near the centre of each leaflet. Sections cut to 3 *μ*m and 10 *μ*m were used for light microscopy (LM) and SAM examinations, respectively. For LM, sections were observed with haematoxylin-eosin and elastica Van Gieson (EVG) staining to distinguish elastin fibres from collagen fibres.

All human sections were acquired from the Hamamatsu University Hospital archives. This research protocol, using stored samples without a link with patient identities, was approved by the Ethics Committee of the Hamamatsu University School of Medicine. Informed consent was obtained from all living patients or the families of the deceased for autopsy cases. The methods were carried out in accordance with the approved guidelines.

### 2.2. Calculation of Leaflet Thickness and Fibrosa Thickness

To calculate the average AV leaflet thickness, the width of the middle third portion of each leaflet without calcified nodules was measured using the LM sections. To calculate the average thickness of the fibrosa, the width of the fibrosa was measured at a minimum of 6 points in the midportion of the leaflet without calcified nodules using the EVG-stained slide.

### 2.3. Measurement of SOS via SAM

The microscope used for SAM (AMS-50SI) was supplied by Honda Electronics (Toyohashi, Aichi, Japan) and was equipped with a 400-MHz transducer (Supplemental Methods S1). A single pulsed wave was used. The SOS within 2.4 × 2.4 mm^2^ or 1.2 × 1.2 mm^2^ was measured via X-Y scanning. The SOS measurements of 300 × 300 points in each scan were plotted to construct images [11]. Because harder tissues result in larger SOS values, SAM provided data regarding the tissue stiffness [5]. If the entire area of the tissue structure exhibited high or low SOS values, it is regarded as having stiff or elastic properties, respectively. Each SAM image included a vacant area that measured the SOS through only water at 1485 m/s as a control area. The SOS values from at least six different points on the fibrosa of each image were collected and analysed statistically.

The resolution of the 400-MHz transducer was approximately 3.75 *μ*m. Unstained flat 10 *μ*m sections were used for scanning. A detailed explanation of the methods is available in the supplemental information (Supplemental Figures 1 and 2).

### 2.4. SOS after Collagenase or Pepsin Digestion

Deparaffinized sections were soaked in collagenase (Sigma C-5138, 450 units/mL in phosphate buffered saline with 0.5 mM CaCl_2_ at pH 7.4) or pepsin (Sigma P-6887, 12 units/mL in 10 mM HCl) solutions at 37°C for 1 or 3 h, respectively. The same sections were used for repeated digestion. After digestion, the sections were washed in distilled water and observed via SAM.

### 2.5. Immunohistochemical Analysis

IHC studies were performed using a commercially available ChemMate Envision kit (Dako, Glostrup, Denmark). The primary antibodies used to detect collagen types, advanced glycation end-products (AGEs), and LOX were as follows: (1) anticollagen I (ab88147, Abcam, Tokyo, Japan; at 1 : 100 concentration), (2) anticollagen III (ab7778, Abcam, Tokyo, Japan; at 1 : 1000 concentration), (3) anti-AGE (AGE102-0.2, Biologo, Kronshagen, Germany; at 1 : 400 concentration), (4) anti-N^*ε*^-carboxymethyllysine (CML) (LifeSpan BioSciences, Seattle, WA; at 1 : 400 concentration), and (5) anti-LOX (ab174316, Abcam, Tokyo, Japan; at 1 : 300 concentration). Heat-mediated antigen retrieval (95°C, 20 min) was performed for the sections with either pH 6.0 (anti-AGE, anti-CML, and anticollagen III) or pH 9.0 (anticollagen I, anti-LOX) buffer prior to IHC staining.

### 2.6. Statistical Analysis

Prior to the statistical tests mentioned below, we confirmed the normal distribution using a test for the difference between means. One-way ANOVA was used to evaluate the thickness of AVs, the thickness of fibrosa, the SOS through fibrosa, and the SOS after protease digestion. Significant differences were evaluated based on multiple comparisons using Tukey-Kramer's test, with *P* < 0.05 considered statistically significant.

## 3. Results

### 3.1. Histological Images of AS and AR Valves and Measurements of the Thickness of Entire Leaflets or Fibrosa

**Figure 1:**
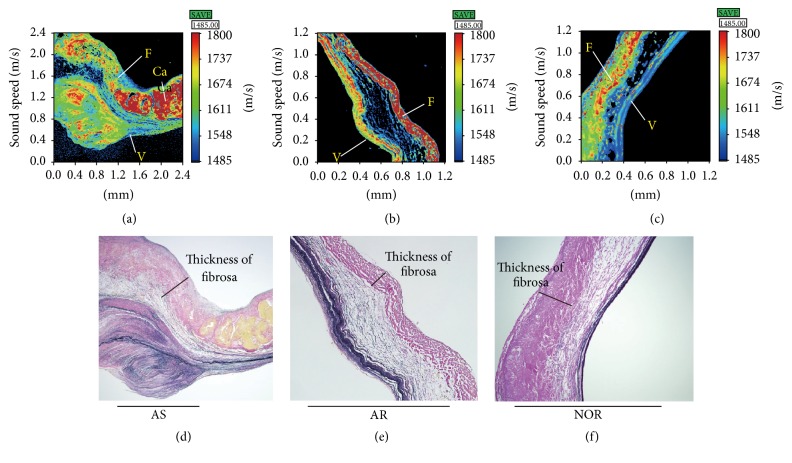
Speed of sound images from scanning acoustic microscopy and the corresponding light microscopic images with elastica Van Gieson staining. Speed of sound (SOS) image of a representative AS case (a) showing thick fibrosa (indicated by “F”) with calcification (“Ca”) and thrombus-formed ventricularis (“V”). The thick fibrosa revealed variable SOS values, except in the nodular calcified areas, which corresponded to the highest SOS values. SOS image of a representative AR case (b) displaying a thin AV composed of a regular, three-layered structure. The fibrosa corresponded to a high SOS value, and the ventricularis (almost the same thickness) displayed a higher SOS than that in the AS group. SOS image of a representative normal (NOR) AV case (c) displaying a three-layered structure with thin, regular ventricularis. Light microscopy (LM) of AS (d) showed atherosclerosis on the fibrosa with an irregular array of collagen fibres. The elastic lamina of the ventricularis was interrupted and protruded into the endothelial surface. The fibrosa in AR visualized via LM (e) consisted of a regular array of thick collagen fibres. The fibrosa of a normal aortic valve (f) occupied approximately half of the total thickness. Scale in LM = 1 mm.

**Figure 2:**
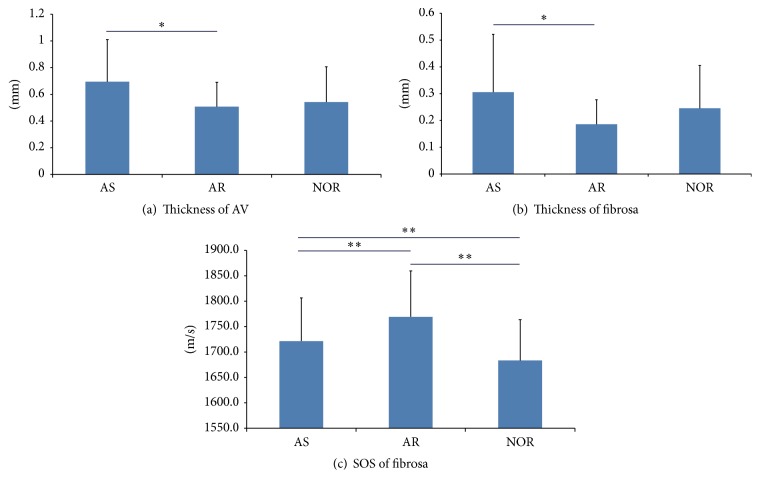
Differences among the AS, AR, and normal (NOR) groups in the total thickness of AVs, the thickness of the fibrosa, and the SOS through the fibrosa. Values represent the mean and SD (*n* = 27, 24, and 36 for the AS, AR, and NOR groups, resp., for the AV and fibrosa thicknesses; *n* = 284, 176, and 318 for AS, AR, and NOR groups, resp., for the SOS through the fibrosa). (a) The total thicknesses of AVs were significantly different (^*∗*^ *P* < 0.05, Tukey-Kramer test) between the AS and AR groups. (b) The thicknesses of the fibrosa also significantly differed (^*∗*^ *P* < 0.05, Tukey-Kramer test) between the two groups. (c) The mean SOS values through the fibrosa were significantly different (^*∗∗*^ *P* < 0.01, Tukey-Kramer test) in each pair of groups.

Although there were no significant differences in the thicknesses of whole AVs and the fibrosa layer between normal and AS groups or between normal and AR groups (Figures 1(a)–1(f), 2(a) and 2(b)), the AS group had significantly thicker leaflets and fibrosa (*P* < 0.05) than the AR group (Table 1). In AS, the fibrosa occupies 45% of the thickness of the whole valve (Figures 1(a) and 1(d)). On the contrary, in AR, the fibrosa occupies 36% of the thickness of the whole valve (Figures 1(b) and 1(e)). The ventricularis layer is distinct even in AR but indistinct and variable in AS, which made it difficult to measure its thickness. The SOS images of AS valves showed thick fibrosa focally with nodular calcification and subendothelial thrombus, and the light microscopic images showed atherosclerosis on the fibrosa with an irregular array of collagen fibres. The elastic lamina of the ventricularis was interrupted in AS. Inflammatory and interstitial cells (ICs) were dense under the subendothelial layer and patchy on the fibrosa and spongiosa in AS.

The SOS images of the AR group revealed thin leaflets composed of a regular three-layered structure. The fibrosa consisted of thick regular collagen fibres forming a regular thin layer. The ventricularis had almost the same thickness as the fibrosa of the AR group. Cellular infiltrate due to inflammatory cells or ICs was rare in the AR group.

### 3.2. SOS Differences among the Normal, AS, and AR Groups

The average SOS through the fibrosa of the three groups increased in the following order: normal group < AS group < AR group (Figure 2(c), Table 1). The AS group displayed significantly higher values (*P* < 0.01) than the normal group. The AR group presented higher values (*P* < 0.01) compared with the AS and normal groups. High SOS values were observed for calcified nodules in valves from AS cases, although the other areas, including atherosclerosis or lipid deposition portions, exhibited variable lower values. The SOS of the fibrosa in AR showed homogenously higher values (*P* < 0.01) for the collagen fibres (Figure 1(b)). The SOS of the ventricularis in the AR group also exhibited uniformly higher values compared with that in the AS group.

### 3.3. Susceptibility to Collagenase and Pepsin

**Figure 3:**
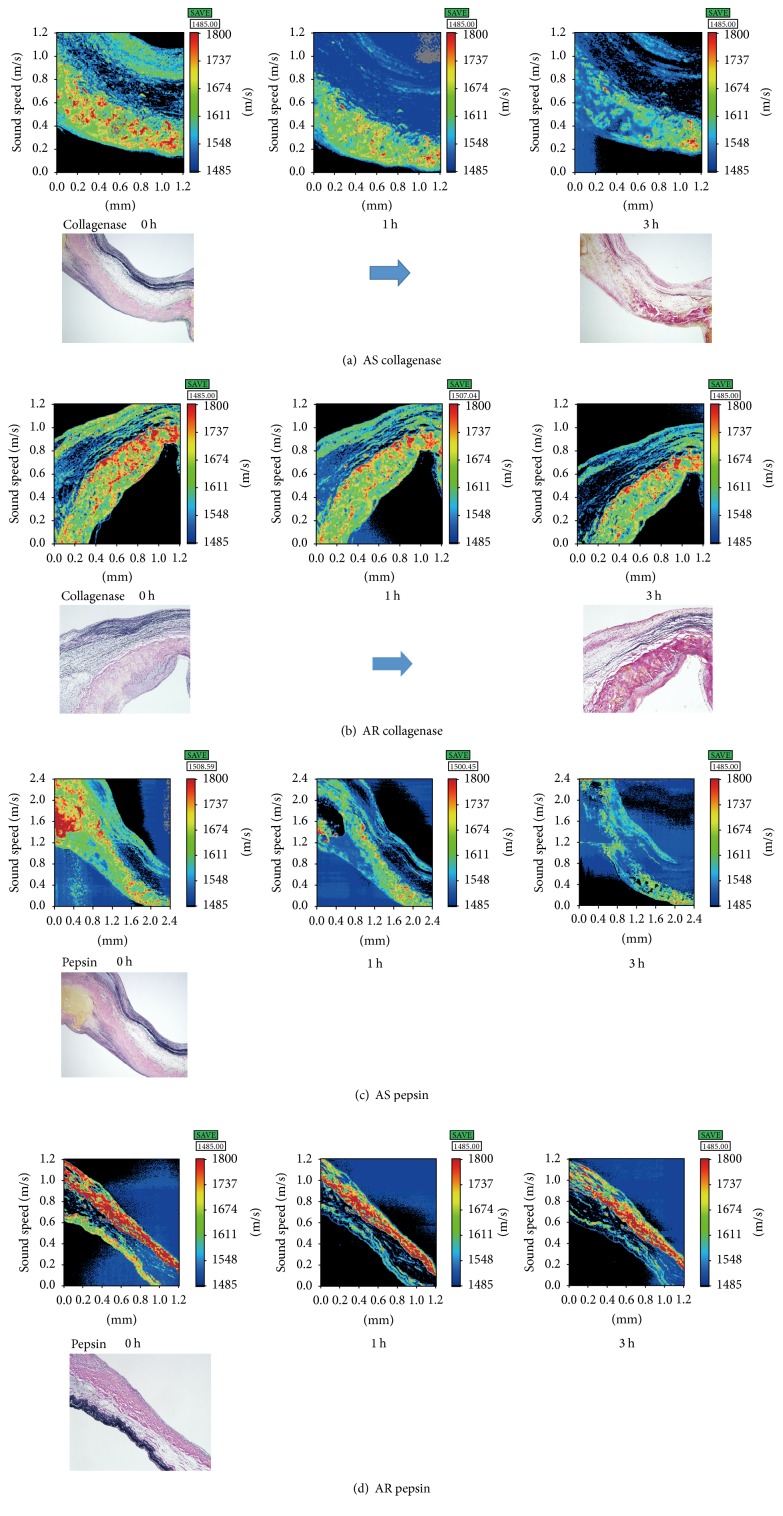
Alteration in speed of sound images after protease digestion and the corresponding LM images with elastica Van Gieson staining. Speed of sound (SOS) image of an AS case (a) showing a rapid decrease in the fibrosa values for the following collagenase digestion. (b) SOS image of an AR case revealing stable SOS values of the fibrosa. SOS image of an AS case after pepsin digestion (c), which also shows a rapid decrease in the SOS values compared to the AR case (d).

**Figure 4:**
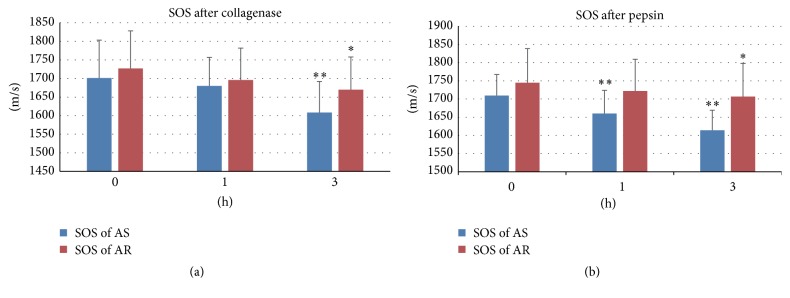
Speed of sound change after protease ((a) collagenase, (b) pepsin) digestion. Values represent the mean and SD (*n* = 30 and 30 points for the AS and AR groups of (a), resp., *n* = 50 and 70 points for the AS and AR groups of (b), resp.). ^*∗∗*^, ^*∗*^Significantly different from the SOS values at 0 h (^*∗∗*^ *P* < 0.01, ^*∗*^ *P* < 0.05; Tukey-Kramer test). Precise data are available in the Supplementary Table  S2 in Supplementary Material available online at http://dx.doi.org/10.1155/2016/6125204.

The SOS values of the fibrosa of the AS and AR groups were significantly decreased (*P* < 0.01, 0.05, resp., Tukey-Kramer test) 3 h after collagenase digestion compared with the SOS values at 0 h (Figures 3(a), 3(b), and 4(a)). Upon pepsin digestion, the SOS values of the fibrosa in the AS group significantly decreased from 1 h to 3 h after digestion (*P* < 0.01) compared with those at 0 h (Figures 3(c), 3(d), and 4(b)). The AR group displayed a significant decrease (*P* < 0.05) only at 3 h after digestion. The AS group exhibited more susceptibility to protease digestion than the AR group.

In the AS group, the elastic fibres of the ventricularis almost disappeared after 3 h of digestion. However, a portion of elastic fibres in the AR group remained.

### 3.4. Immunohistochemical Analysis

**Figure 5:**
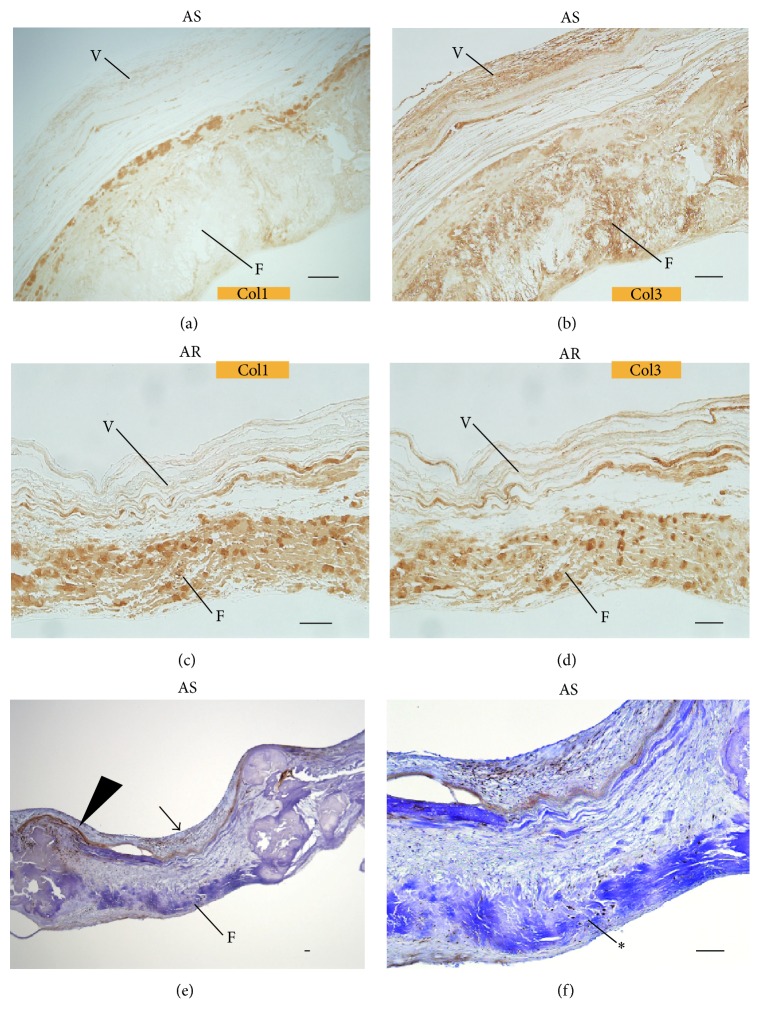
Immunostaining with anticollagen type I or III and anti-LOX. In the AS group (upper row), collagen type I (a) was present at the inner side of the fibrosa (marked “F”), and collagen type III (b) was widely distributed in the rest of the fibrosa and ventricularis (the latter marked “V”). In the AR group (lower row), collagen types I (c) and III (d) coexisted in the fibrosa and ventricularis. ((e), (f)) The ICs of organized thrombi gathered at subendothelial locations were positive for anti-LOX staining (arrow) and the cells in the fibrosa (star) were positive, especially surrounding calcified nodules (arrowhead). Scale bar = 100 *μ*m.

With regard to collagen types, in the AS group, collagen type I was left focally on the fibrosa, and collagen type III was present throughout the rest of the fibrosa (Figures 5(a)–5(d)). The ventricularis was composed of lamellar elastic fibres predominantly containing collagen type III. In the AR group, types I and III collagen fibres coexisted in the fibrosa and ventricularis.

LOX staining revealed that the ICs in the fibrosa of the AS group, particularly surrounding calcified nodules, were positively stained, whereas the fibrosa of the AR group was primarily negative (Figures 5(e)–5(f)). ICs in organized thrombi were strongly positively stained in the AS and AR groups.

**Figure 6:**
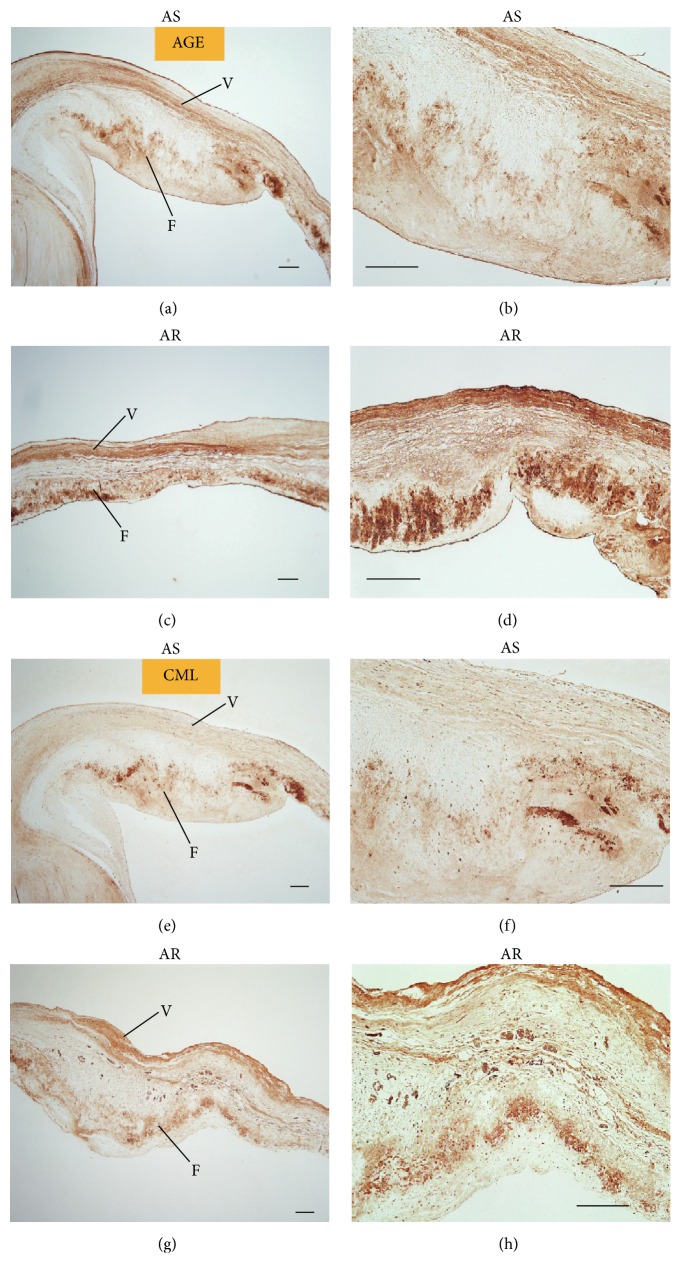
Immunostaining with antiadvanced glycation end-products (AGEs). Upon immunostaining the AS or AR valves with anti-AGE ((a)–(d)) and anti-CML ((e)–(h)) antibodies, the AS group ((a), (b), (e), and (f)) showed sparse dot-like staining of the fibrosa (F), whereas the AR group ((c), (d), (g), and (h)) presented a strong band-like positive reaction on the fibrosa. The ventricularis (marked “V”) of the AR group also demonstrated a lamellar-positive reaction on the elastic fibres compared with faint staining of the AS group. Short scale bar = 200 *μ*m; long scale bar = 100 *μ*m.

AGE staining using anti-AGE and anti-CML antibodies revealed that the AS group showed sparse, weak staining of the fibrosa, whereas the AR group showed a strong band-like positive reaction in the fibrosa (Figure 6). The ventricularis also demonstrated a lamellar-positive reaction in the elastic fibres of the AR group, whereas weak or faintly positive staining was observed on the ventricularis of the AS group.

## 4. Discussion

Chemical alterations associated with morphological changes were revealed by the SOS, which followed protease digestion and IHC to detect antigen location.  Table 2 summarizes the characteristic differences between the two forms of AV ageing. The AS group is characterized by thick nodular leaflets with active reparative changes, such as fibrosis, atherosclerosis, and calcification. Type I collagen in the fibrosa effectively disappeared and was replaced by type III collagen in the AS group. The deposition of AGEs was slight in AS. However, the AR group was composed of thin leaflets with a regular three-layered structure. Active reparative changes were rare in this group, and the deposition of AGEs was diffuse and conspicuous, showing strong resistance to protease digestion.

The AS group was more susceptible to collagenase or pepsin digestion than the AR group. The fibrosa of the AR valves was harder than the fibrosa of the AS valves because the collagen in the former had longer life spans with more cross-linking or alterations. The turnover of collagen should be slower in the AR group compared with the AS group because ICs, which produce collagen, and LOX were more sparse in the AR group compared to the AS group. The AS group showed more inflammatory cell infiltrates with dense ICs. These new collagen fibres were more susceptible to protease digestion than older collagen fibres.

Collagen types I and III fibres usually coexist in the fibrosa and ventricularis [3] in normal valves, as observed in the AR group. In the regenerative process of wound repair, fine collagen type III fibres are initially formed following collagen destruction, which are gradually replaced by type I collagen fibres [16]. The AS group primarily showed type III collagen fibres, except at the internal side of the fibrosa. Inflammatory cell infiltrate within the thrombi was more conspicuous near endothelial cells compared with deeper areas, resulting in more collagen remodelling at surface areas.

Collagen accounts for 50% of the total protein in noncalcified regions and 10% in calcified regions, compared with 90% of the total protein in healthy valves [17]. The concentrations of types I and III collagen decrease during ageing [17]. The decrease in total collagen content, despite the increase in type I collagen mRNA expression, indicates an increase in collagen turnover in AS. Comparing elderly AS valves with infectious endocarditis (IEC) valves, both types of thick valves exhibit different vascular proliferation reactions. IEC valves contain many capillaries accompanied with various inflammatory cells, whereas AS valves contain few capillaries with few inflammatory cells. Compared to the activity of myofibroblasts in both valves, myofibroblasts in IEC valves produce many collagen fibres, including thick type I collagen in granulation tissues, whereas those in AS vales produce thin collagen type III fibres but not type I fibres. Blood supply shortage causes myofibroblasts to have short lifespans for producing mature type I collagen.

Human ageing is characterized by decreased turnovers of collagen and elastin and increased AGEs and cross-links between fibres [18]. Elastic fibres undergo lysis and disorganization subsequent to their replacement by collagen and other matrix components. These events cause a loss in elasticity and induce stiffening.  Figure 2 and Table 1 show that diseased fibrosa of AS and AR values has significantly greater SOS values, implying that the diseased fibrosa composed of collagen fibres is significantly stiffer than normal fibres. In our immunohistochemical study, many AGEs were deposited on collagen and elastin, especially in AR cases, inducing resistance to protease digestion. Elastin in the ventricularis in the AR group exhibited fine lamellar fibre structures with larger SOS values compared with those of the AS group, suggesting that the elastin of the AR group was also stiffer than that of the AS group.

Anti-LOX staining revealed that positive fibroblasts were rare in AR cases. These results indicate that new synthesis of collagen and elastin reduces and that accumulation of old fibers increases on the contrary.

In contrast, the AS group showed few AGEs with greater sensitivity against protease digestion. The elastin of the ventricularis and the collagen of the fibrosa were easily digested by proteases. Anti-LOX positive ICs were scattered in the valves. LOX exerts copper-dependent amine oxidase activity and functions in the cross-linking of collagens and elastin [19]. These results suggest that the collagen and elastin in AS cases are newly produced and stabilized.

Nonenzymatic glycosylation (or Maillard reaction) of proteins primarily takes place at *ε*-amino groups of lysine or their free amino groups [15]. Amadori-modified proteins, an early glycation product, undergo further reactions through a number of pathways, giving rise to AGEs [20]. Glycation has been used to explain one of the mechanisms of ageing [21]. According to glycation theory, intermolecular cross-linking and the denaturation of proteins caused by glycation are major factors of early ageing-induced alterations in tissues and blood vessels [22, 23]. These cross-links decrease critical flexibility and reduce protein turnover [24].

Recent data suggests that calcific AV disease is an active cellular process that develops within valve leaflets [25, 26]. Mechanical stress on the AV and atherosclerosis risk factors lead to valvular endothelial dysfunction and leakage, followed by the deposition of lipids and other compounds. These events trigger inflammation, which in turn activate valvular myofibroblasts resulting in their osteoblastic transdifferentiation. The present study supports this pathological process of AS, and inhibiting active inflammatory and regenerative processes may prevent the progression of AS.

To date, there is little information regarding the mechanical properties of diseased AVs due to the need of relatively large tissue samples for standard mechanical test methods [3]. Atomic force microscopy [27] or microaspiration [28] is used in biophysics to measure the mechanical properties of living materials. The microaspiration method estimates cell stiffness by measuring the degree of membrane deformation in response to the negative pressure applied by a glass micropipette to the cell surface. Although SAM cannot scan living cardiac valves, it can utilize the same histologic sections for routine diagnoses and detect chemical modifications to structural proteins after enzymatic digestion. Increased resolution is possible by adjusting the scan area or by increasing the frequency of the ultrasound. Using SAM, we have been able to detect single cells and even cell nuclei [29].

The total valve elasticity depends on the SOS values of the three layers in addition to the ratio and connection of these layers. In thick AS valves with calcification, the fibrosa occupies almost half of the total valve thickness. In contrast, in thin AR valves without calcification, the fibrosa occupies only one-third of the entire thickness. The borders among the layers are indistinct in AS but distinct in AR. The width of the spongiosa in AS is variable due to the infiltration of collagen or elastic fibres, whereas that in AR is constantly maintained. Proteoglycans in the spongiosa function as a cushion for absorbing tension and friction. Thus, the total properties of elasticity are stiff in AS and elastic in AR.

Regarding the examined samples, we used routine formalin-fixed, paraffin-embedded (FFPE) tissues for this study. Compared with unfixed tissues, formalin fixation induces tissue stiffness due to cross-links among proteins, which is detected by increased SOS values [8]. In our preliminary study, the effects of formalin fixation on SOS alterations in AV were evaluated after fixation of fresh frozen sections. There was no remarkable elevation in the SOS up to 24 h. After 24 h, the SOS values of various organs were stable for up to 3 months [8]. The postoperative fixation period of resected valves in this study was between 1 and 3 days. For normal valves taken from autopsies, the fixation period used was longer, lasting up to a few months. Comparing the SOS data among the three groups, normal AVs from autopsies had the lowest SOS values, which indicates that normal AVs that underwent longer fixation periods were more elastic than AS or AR valves that underwent short fixation periods. In this study, the fixation and tissue processing treatments did not significantly affect the results. The presented values may, therefore, have close similarities to those possessed by fresh valves in vivo.

## 5. Conclusions

In conclusion, we showed that AS and AR valves have distinct structural differences related to the ageing process. AS valves display tissue remodelling upon damage and repair, whereas AR valves exhibit static chemical alterations due to ageing with slow collagen turnover. This study using both SAM and immunohistochemical methods revealed correlations between biomechanical and biochemical alterations that provides a novel tool for studying mechanism(s) associated with AV diseases.

## Supplementary Material

Supplemental Methods S1 Observation method by scanning acoustic microscopy (SAM) was explained.

## Figures and Tables

**Table:** (a) Sample aortic valves

AV	M	F	AGE	Range
AS	9	9	72.3 ± 12.1	60–84
AR	9	5	68.1 ± 7.4	53–80
NOR	10	5	72.7 ± 14.9	50–101

**Table:** (b) AV thickness

AV thickness	*n*	Mean (*μ*m)	*U*	SD	SE
AS	27	694	100	316	61
AR	24	508	33	182	37
NOR	36	542	70	264	44

**Table:** (c) Fibrosa thickness

F thickness	*n*	Mean (*μ*m)	*U*	SD	SE
AS	27	306	47	216	42
AR	24	186	8	91	19
NOR	36	245	26	160	27

**Table:** (d) Speed of sound through the fibrosa

SOS of F	*n*	Mean (m/s)	*U*	SD	SE
AS	284	1721.5	7242.6	85.1	5.0
AR	176	1769.2	8196.7	90.5	6.8
NOR	318	1683.3	6454.4	80.3	4.5

The sample aortic valves (AVs) of elderly patients (a) were divided into AS, AR, and normal groups. The thicknesses of the AVs (b) and fibrosa (c) and the speed of sound (SOS) values (d) through the fibrosa are reported as the means, the unbiased estimate of population variances (*U*), the standard deviations (SD), and the standard errors (SE). The AV width was measured at the middle portion of each leaflet. The SOS was measured through the fibrosa without calcification of each leaflet. Significant differences were observed in the total AV thickness and the thickness of the fibrosa between the AS and AR groups (*P* < 0.05, Tukey-Kramer test). The SOS through the fibrosa was significantly different in each group (*P* < 0.01, Tukey-Kramer test). Analysis of variance tables is shown in supplementary Table S1 online. M, male; F, female; F, fibrosa.

**Table 2:** Characteristic differences between AS and AR.

AV	Calc/lipid	F. array	Capillary	IC/inf cells	COL type	COLase	AGEs deposit	LOX
AS	Many	Irregular	Many	Many	1 & 3 separate	Sens	Sparse	Pathy
AR	A few	Regular	A few	A few	1 & 3 coexist	Resist	Diffuse	Rare

Calc, calcification; F. array, fibre array; IC/inf cells, interstitial cell/inflammatory cells; COL type, collagen type; COLase, collagenase; AGEs, advanced glycation end-products; LOX, lysyl oxidase.
