# Supplementary material for: Structural and Histochemical Alterations in the Aortic Valves of Elderly Patients: A Comparative Study of Aortic Stenosis, Aortic Regurgitation, and Normal Valves
Source: Biomed Res Int. 2016 Sep 25;2016:6125204. doi: 10.1155/2016/6125204 (PMC5056004; doi:10.1155/2016/6125204)
Supplement: Supplementary file 1 — Supplemental Methods S1 Observation method by scanning acoustic microscopy (SAM) was explained. [file 6125204.f1.pdf]

SUPPORTING INFORMATION

Supplemental Methods S1

Observation by scanning acoustic microscopy

The scanning acoustic microscopy (SAM) used a single pulsed wave to image objects. SAM functions by directing focused sound from a transducer to a small area of the target object on a glass slide (Supplemental Figure 1). The sound emitted by an acoustic transducer hits or penetrates the tissue and is reflected onto the surface of the tissue or glass. It is then returned to the detector, which is coincident with the transducer. The speed of sound (SOS) through the tissue is calculated by comparing the "time of flight" of the pulse from the surfaces of both tissue and glass. Each specimen was scanned in a frame of 300 × 300 pixels, and an area 1200 × 1200 μm^2 was scanned to obtain each image within 3 min. The two-dimensional distribution of the echo intensity of sound, SOS, attenuation of sound (AOS) through the tissues, and the thickness of the tissue was obtained and plotted (Supplemental Figure 2). The penetration depth was up to a maximum of 20 μm. The observation range within which the values were plotted could be adjusted.

Supplemental Figure 1

Principle of a scanning acoustic microscope

The ultrasound emitted by an acoustic transducer hits or penetrates the tissue and is reflected onto the surface of the tissue or glass slide. It is then returned to the detector. The speed of sound through the tissue is calculated by comparing the "time of flight" of the pulse from the surfaces of both the tissue and glass.

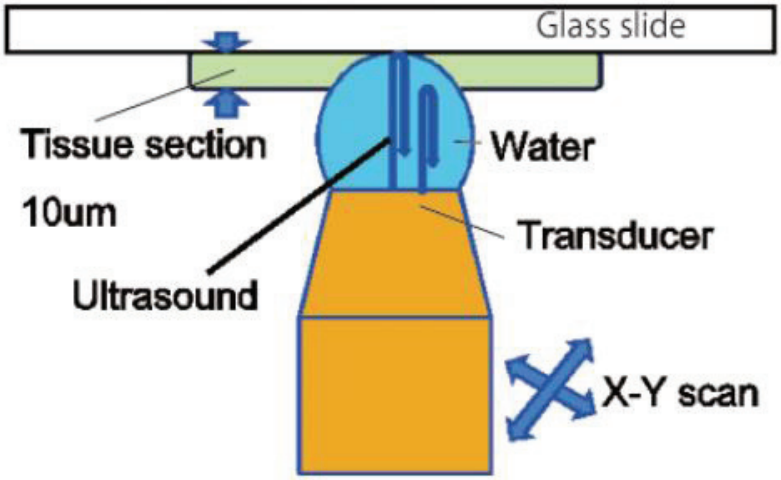

Supplemental Figure 2

SAM images of intensity (A), sound speed (B), attenuation (C), and thickness (D) of the tissue

On the screen, the vertical bar at the left side and the horizontal bar at the bottom of each figure indicate the distance (mm) on the slide (arrows).

The vertical colour column at the right side of the "Sound-Speed" figure (upper right image indicated by *) indicates the average SOS of each square area on the section. Similarly, the vertical colour columns in "Attenuation" (lower left image) and "Thickness" (lower right image) indicate the attenuated intensity-of-sound per mm (dB/mm) and the thickness of each square area (μ m), respectively.

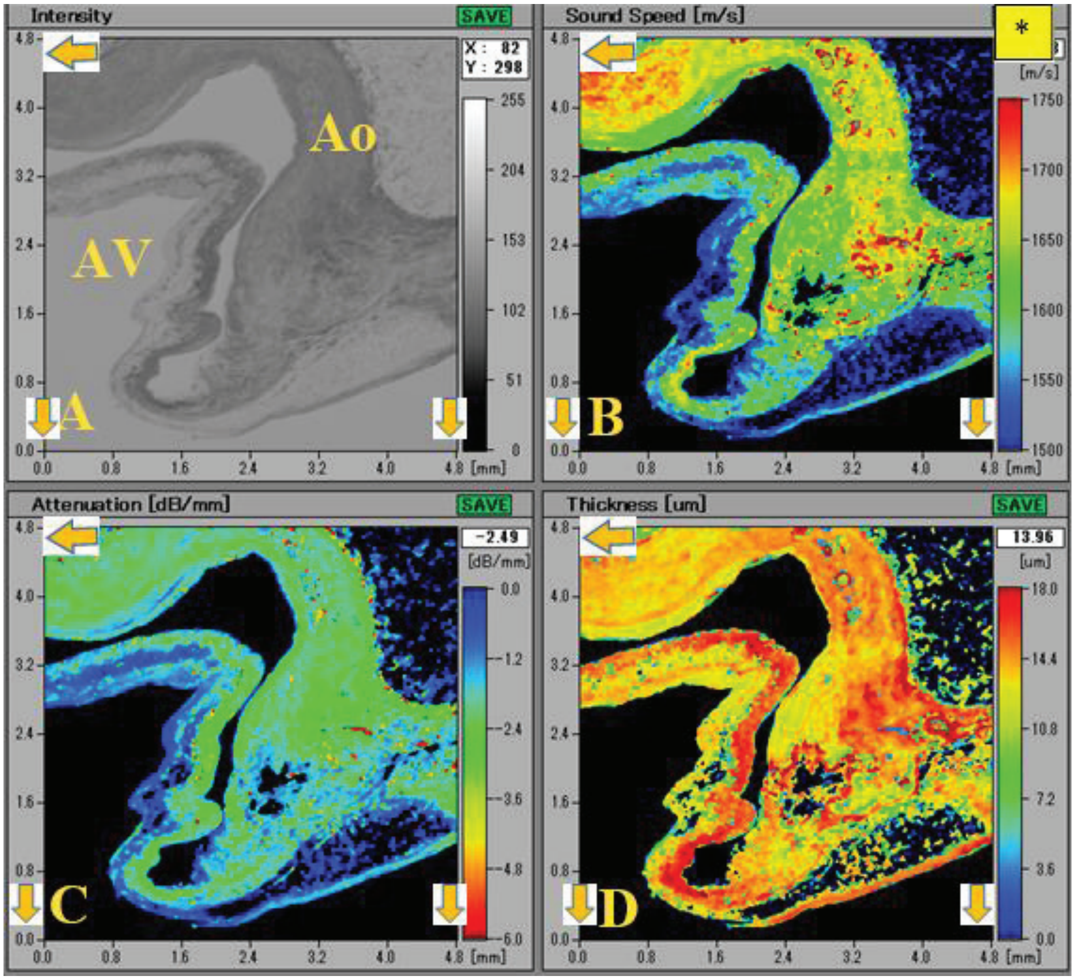
Intensity
SAVE
X : 82
Y : 298
Ao
AV
A
255
204
153
102
51
0
Sound Speed [m/s]
*
[m/s]
1750
1700
1650
1600
1550
1500
B
Attenuation [dB/mm]
SAVE
-2.49
[dB/mm]
0.0
-1.2
-2.4
-3.6
-4.8
-6.0
C
Thickness [um]
SAVE
13.96
[um]
18.0
14.4
10.8
7.2
3.6
0.0
D
4.8
4.0
3.2
2.4
1.6
0.8
0.0
[mm]

Supplementary Table S1

Analysis of variance table (AV thickness, Fibrosa thickness, SOS through fibrosa)

AV thickness

Source	SS	dF	MS	F-statistic	P-value	F (0.95)	F (0.99)
Total variation	6.32	86					
Between group	0.53	2	0.265	3.84	0.0254	3.105	4.867
Within group	5.79	84	0.069				

Fibrosa thickness

Source	SS	dF	MS	F-statistic	P-value	F (0.95)	F (0.99)
Total variation	6.32	86					
Between group	0.53	2	0.265	3.84	0.0254	3.105	4.867
Within group	5.79	84	0.069				

SOS through fibrosa

Source	SS	dF	MS	F-statistic	P-value	F (0.95)	F (0.99)
Total variation	6375805	777					
Between group	845685	2	422842.6	59.26	1.13E-24	3.007	4.633
Within group	5530120	775	7135.6				

SS, sum of squares; dF, degrees of freedom in the source; MS, mean sum of squares due to the source.

Supplementary Table S2

SOS after protease digestion

SOS after collagenase (AS cases)

AS collagenase	n	Mean (m/s)	U	SD	SE
0 h	30	1701.0	10408.7	102.0	18.6
1 h	30	1679.7	5949.7	77.1	14.1
3 h	30	1608.5	7015.6	83.8	15.3

Analysis of variance table (ANOVA Table) (AS cases)

Source	SS	dF	MS	F-statistic	P-value	F (0.95)	F (0.99)
Total variation	818755.5	89					
Between group	140909.6	2	70454.8	9.043	0.00027	3.10	4.86
Within group	677845.9	87	7791.3				

SOS after collagenase (AR cases)

AR collagenase	n	mean(m/s)	U	SD	SE
0 h	30	1727.0	10228.6	101.14	18.5
1 h	30	1695.6	7396.4	86.00	15.7
3 h	31	1669.9	7748.5	88.03	15.8

ANOVA Table (AR cases)

Source	SS	dF	MS	F-statistic	P-value	F (0.95)	F (0.99)
Total variation	793345.1	90					
Between group	49765.01	2	24882.5	2.94	0.058	3.10	4.85
Within group	743580.1	88	8449.8				

SOS after pepsin (AS cases)

AS Pepsin	n	mean(m/s)	U	SD	SE
0 h	50	1709.4	3350.7	57.9	8.19
1 h	51	1660.0	4048.2	63.6	8.91
3 h	51	1614.1	2999.6	54.8	7.67

ANOVA Table (AS cases)

Source	SS	dF	MS	F-statistic	P-value	F (0.95)	F (0.99)
Total variation	745815.7	151					
Between group	229241	2	114620.5	33.1	1.31E-12	3.06	4.75
Within group	516574.6	149	3466.944				

SOS after pepsin (AR cases)

AR pepsin	n	mean(m/s)	U	SD	SE
0 h	71	1745.0	8769.1	93.6	11.1
1 h	70	1722.1	7573.6	87.0	10.4
3 h	72	1706.7	8324.5	91.2	10.8

ANOVA Table (AR cases)

Source	SS	dF	MS	F-statistic	P-value	F (0.95)	F(0.99)
Total variation	1780673	212					
Between group	53223.52	2	26611.8	3.24	0.041	3.04	4.71
Within group	1727450	210	8226.0				

U, unbiased estimate of population variance; SS, sum of squares; dF, degrees of freedom in the source; MS, mean sum of squares due to the source.
